# Visualizing the change of “viewpoints” in 3D virtual art exhibition

**DOI:** 10.1177/20416695251314182

**Published:** 2025-01-31

**Authors:** Kazuki Matsumoto, Takeshi Okada

**Affiliations:** Dokkyo University, Japan; 13143 The University of Tokyo, Japan

**Keywords:** art viewing, methodological research, non-immersive virtual reality, movement, Bayesian hierarchical analysis, mode of viewing

## Abstract

This study focuses on the process of viewers’ engagement with artwork in virtual spaces. Specifically, we propose a methodology that combines two approaches: (a) measuring movements within virtual spaces, and (b) using non-immersive virtual reality. We attempt to demonstrate the effectiveness of this methodology through experimentation. In the experiment, we recorded the exploration of sculptures in virtual spaces of 317 participants using a newly developed system (Virtual Exhibition Space for Tracking and Analyzing system). Results from multiple statistical analyses, including hierarchical Bayesian analysis, indicated differences in view-angles and distances traveled while viewing the artworks under different experimental conditions (where different instructions were given prior to viewing). These findings support the theoretical expectation that the “mode” of viewing artworks is influenced by instructions given before viewing, which is further corroborated by the observed differences in the pattern of psychological measurements between conditions. Based on previous research and these experimental results, we discuss the effectiveness and generalizability of our methodology in capturing variations in the art-viewing process as a tool for studying art-viewing behavior not only in virtual but also in real environments.

## How to cite this article

Matsumoto, K., & Okada, T. (2025). Visualizing the change of “viewpoints” in 3D virtual art exhibition. *i-Perception*, *16*(0), 1–21. https://doi.org/10.1177/20416695251314182

How do people view artworks in virtual spaces? Art researchers have found value in exploring this question due to the rapid rise in virtual art experiences in recent years (Geismar, 2018; Schweibenz, 2019) and because these experiences seem to differ from traditional art encounters. However, previous research on virtual art-viewing has two limitations: (a) it has primarily focused on psychological measures and has not captured viewers’ movements in virtual spaces; and (b) it has predominantly considered immersive virtual reality (VR) as virtual research and has not sufficiently explored non-immersive VR (desktop VR). In the following sections, we offer an overview of the significance of these two issues. Thereafter, we discuss the research questions that this method is suited to address.

Through theoretical considerations and experimentation, this study aims to highlight the effectiveness of a research design that simultaneously addresses these two aspects. As later elaborated, this methodology is expected to significantly contribute not only to understanding virtual art experiences but also to the general comprehension of art experiences or other research topics.

### Movements in Virtual and Non-Virtual Experience of Art

The art experience involves physical as well as mental processes, even if it refers to the “passive” recognition of artworks, such as viewing paintings or listening to music. This intuitively makes sense if the same artwork can give a considerably different impression depending on whether one examines it with one's eyes close or views it from a distance. In a non-virtual context, previous studies have already examined how the body affects the artwork's impression. For example, experiments have confirmed that hand movements (Leder et al., 2012; Ticini et al., 2014), eye movements (Matsumoto & Okada, 2021a), and hands-on creative activities (Matsumoto & Okada, 2021b) affect viewers’ esthetic impression. In a broader sense, these findings are closely related to recent theories, such as those of Carbon (2019, 2020), which emphasize what kind of physical context an artwork is viewed within. This is because the bodily state, also expressed as “bodily context,” can be interpreted as one of the physical contexts that may generally affect one's judgment or feelings (cf. Maglio & Trope, 2012; Troncoso et al., 2023).

It is plausible—both empirically and intuitively—that the body influences mental states during art-viewing as an explanatory variable; at the same time, the body may reflect influences from factors within the process of art-viewing, serving as an response variable. Art-viewing is an information-seeking process, and the viewer actively moves their body to acquire information. Even when facing the same artwork, the flow of processed information can be completely different depending on the spatial trajectory of perspectives. This implies that people must adjust their movements according to what they want to pay attention to. Perceptual input information is shaped by movement, which, in turn, is influenced by the results of cognitive processing. As this microcycle of perception and movement is a constant presence in art-viewing, a comprehensive understanding of art-viewing is incomplete without measuring actual movement.

The significance of movement does not diminish in the context of art-viewing in virtual spaces. “Virtual space” here refers to an electronic recreation of actual space that includes single or multiple representations of artworks. In a typical virtual space, viewers can navigate within the virtual environment and manipulate a replica in ways similar to those in the actual space. The objective of virtualization is to simulate experiences as realistically as possible, resembling the encounters one would have with actual artworks in the actual space. The most important similarity between the virtual and actual spaces is that the object provides new perceptual information when we interact with it. For example, we can see the hidden other side of the object by rotating it both within the virtual and actual spaces, which is impossible with a static 2D photograph. This capability of virtual spaces creates a sensation akin to being physically in the presence of the actual object—in other words, a “virtual” sensation. Therefore, not only does the viewers’ movement in virtual spaces serve a function equivalent to that of viewers in actual spaces, but it also holds a higher value for consideration due to its direct influence on the quality of virtuality.

Conducting empirical research on movement in virtual spaces is also a powerful means to examine non-virtual experiences. This is because the behavioral history of individuals in virtual spaces can be digitally recorded, which is much more accurate and convenient than recording the coordinates of people and objects in actual spaces (cf. Yaremych & Persky, 2019). To illustrate this point, we examine the study by Kühnapfel et al. (2024) as a comprehensive attempt to capture the dynamics of movement during art-viewing. They displayed a copy of Franz Marc's painting in a room arranged for the experiment, resembling a gallery, and ushered each participant into the space to freely view the work. Data were collected synchronously using mobile eye-tracking glasses worn by the participants and movement-tracking cameras installed in the room. They conducted a thorough analysis of the participants’ trajectories in the room and their gaze patterns on the artwork and surrounding space. They also examined how these related to psychological measurements (e.g., positive emotions toward the work), later measured by subjective reports. Although preparing an actual room and specialized equipment in this study holds unique significance, creating similar situations in a virtual space could be a feasible and realistic alternative for many researchers. With only a computer and a monitor, one can recreate a virtual exhibition space and record the user's camera trajectory (standing for time-varying perspective) within it. In areas other than art-viewing, studies have increasingly focused on measuring movement in virtual spaces (cf. Shamay-Tsoory & Mendelsohn, 2019; Yaremych & Persky, 2019).

Presumably, the lack of measurement of physical movement in virtual spaces can be attributed to the historical disregard for the relationship between art-viewing and physical states. Given the importance of the aforementioned movement in this context, we propose incorporating movement measurement into a new methodology.

### Types of VR and Art-Viewing

Typically, what is referred to as VR involves the use of specialized equipment, such as head-mounted displays (HMDs), to provide viewers with a heightened sense of immersion. To distinguish this type of VR, the term “immersive VR” is sometimes used. In recent years, the art world has witnessed an increase in VR-based artworks and exhibitions. Consequently, several empirical studies have explored art in immersive VR (e.g., Gotthardt et al., 2025; Janković et al., 2020; Lin et al., 2020; Marín-Morales et al., 2019; Miura & Kawai, 2019). However, the virtual art experience is not limited to immersive VR alone. For example, Leopardi et al. (2021) categorized virtual museums into five groups (also see Beer, 2015; Styliani et al., 2009), of which non-immersive VR (or desktop VR) is, in our opinion, of particular importance. This entails using standard flatscreen devices to enable user interaction and partially simulate the subject's virtual presence, as seen in everyday computer programs such as 3D action games or Google Maps. Despite not being commonly labeled as “VR” in ordinary situations, non-immersive VR is used much more frequently than immersive VR. This is due to its ease of adoption for both users and creators—particularly, its convenience for users to be able to view it on a regular monitor without requiring an HMD (cf. Barbieri et al., 2017; He et al., 2023).

non-immersive VR is valuable in research not only for its prevalence in the art domain but also for its intermediary position between traditional digital images and immersive VR. As most existing art-viewing research uses non-virtual digital images, non-immersive VR, which serves as an intermediary, is likely to play a unique role in integrating insights from this approach with those obtained from VR. Moreover, from a practical standpoint, non-immersive VR systems provide an advantage by enabling participation in experiments without requiring an HMD. This makes experiments accessible to a wider range of people in the context of remote online studies, where people with only a computer and a monitor can join in. In summary, exploring artistic experiences with non-immersive VR is considered highly effective both theoretically and practically.^1^ The details of the non-immersive VR application are described in the method section.

### Mode of Art-Viewing: What the Proposed Methodology Can Reveal

To demonstrate the effectiveness of our methodology, which focuses on motion in virtual spaces and employs non-immersive VR, we experimentally aim to capture changes in art-viewing processes by tracking movements within the non-immersive virtual space. When people view artwork, different kinds of global patterns are identified in their processes, which we term “mode” of viewing. In this section, we discuss the nature of that and how it aligns with our methodology.

When we view an object, where we direct out attention to various elements in it sequentially, it is improbable that the processed element at any moment is determined independent of the moment right before it. Rather, our stream of thought and attention is always directed by some temporary topic in a top-down manner. For example, in front of Leonardo da Vinci's “Annunciation,” someone may be interested in the narrative aspect depicted in it. In this situation, this viewer's mental and physical processes will naturally be involved with elements related to the narrative topic, such as gazing upon the characters’ facial expressions, imagining their conversation and feelings, and so on. We could label this type of process as “narrative mode.” On the other hand, individuals with expertise in the creative process of art may focus on the techniques used and the presence of the artist's originality and imagination, rather than the story, in the painting. This mode of focusing on the artwork's creative process (“process mode”) may involve specific psychological processes not observed in the “narrative mode,” such as the viewer's social comparisons with the artist and associated emotions (cf. Hitsuwari & Nomura, 2023; Matsumoto & Okada, 2021a, 2021b). Conversely, the “narrative mode” should naturally have unique aspects absent in the process mode. Therefore, the “narrative mode” and “process mode” each refer to entirely different psychological processes whose difference comes out of the appreciation of the same artwork. Just as ordinary people have the “narrative mode” and experts the “process mode,” differences in knowledge and experience are crucial for differentiating these modes (cf. Bullot & Reber, 2013; Matsumoto & Okada, 2021a; Parsons, 1987).

In addition to shifts in perspective based on individual differences such as knowledge and experience, different situations and goals of viewing artwork can result in mode variations. Viewing art can serve as an autotelic activity for the viewer, offering self-rewarding feelings; in other cases, however, it may also be undertaken as part of studying for an art history exam. Furthermore, viewers may also look at others’ creations to find inspiration for their own new works. The examples here suggest that the way art is viewed can be influenced not only by individual differences but also by the context; thus, the same person may switch modes depending on the time and circumstances (this point will be revisited when experimentally manipulating modes later).

Physical movement patterns play an essential role in distinguishing modes of viewing. If viewers have different interests, such as the depicted characters’ emotions or the painting's creative process, the location of valuable information for them also varies accordingly, such as the characters’ expressions or the texture of paint and brushstrokes across the entire canvas. Thus, viewers can exhibit the differences in the physical distances and angles they prefer during information seeking and their trajectories over time. This aligns with the results of many studies on gaze behavior during art-viewing, which indicates that eye-movement patterns vary based on the viewers’ interests (for review, see Rosenberg & Klein, 2015). In short, the difference in modes of viewing is closely tied to the spatial aspects of viewer's behavior. What has been discussed here echoes the earlier discussion about the general importance of movement in the information-seeking process of viewing artworks. This is the reason why focusing the concept of “mode” of viewing is suitable for the current methodology featured by measuring movements.

### Comparison of Modes: Current Hypotheses

In this study, we aim to capture changes in modes of viewing using a new methodology. To achieve this, we first generate variations in modes through experimental manipulation. Hence, we plan to guide individuals to perceive the same artwork differently using instructional cues. What we focus on are the three modes mentioned in the above discussion: the “Narrative Mode,” “Process Mode,” and “Creator Mode.” The “Narrative Mode” and “Process Mode” refer to states where attention is particularly focused on either the narrative aspects represented in the artwork or the creative process aspects, respectively. In this study, we attempt to evoke these two modes by providing viewers with relevant information on the artwork. By contrast, the third condition, “Creator Mode,” may occur independently of knowledge about the artwork. It is included in this comparison with the expectation of supporting the aforementioned prediction that not only individual differences (such as knowledge) but also the mode changes based on the external context. The “Creator Mode” involves viewing art with the goal of creating one's own works, rather than simply viewing the artwork itself.

As a hypothesis, we anticipate differences in the patterns of scores for psychological variables (e.g., liking, admiration, and interest) in response to the artwork under each condition. Since different modes are likely to influence an entire pattern of the viewing process, we consider using a multidimensional and comprehensive scale and conducting multivariate analysis of variance (MANOVA) suitable for examining the differences. In addition, the appreciation of artworks focusing on the creative process may yield higher scores for both liking and admiration (Matsumoto & Okada, 2021a, 2021b). This suggests the possibility of higher scores of them in the “Process Mode” in this study. More unique to our methodology, we expect differences in information search movements corresponding to each mode—specifically in the distance moved and view-angles when viewing artwork in the virtual space.

## Method

### Participants

A total of 349 participants participated in this study. Due to various issues, such as browser crashes, 32 participants failed to complete the experiment, and data from 317 participants were available for analysis. We recruited the participants online through a crowdsourcing service and gave them approximately JPY 700 after completing the procedure. All methods and procedures were approved by the Ethics Committee of the University of Tokyo.

### Stimuli

**Figure 1.**
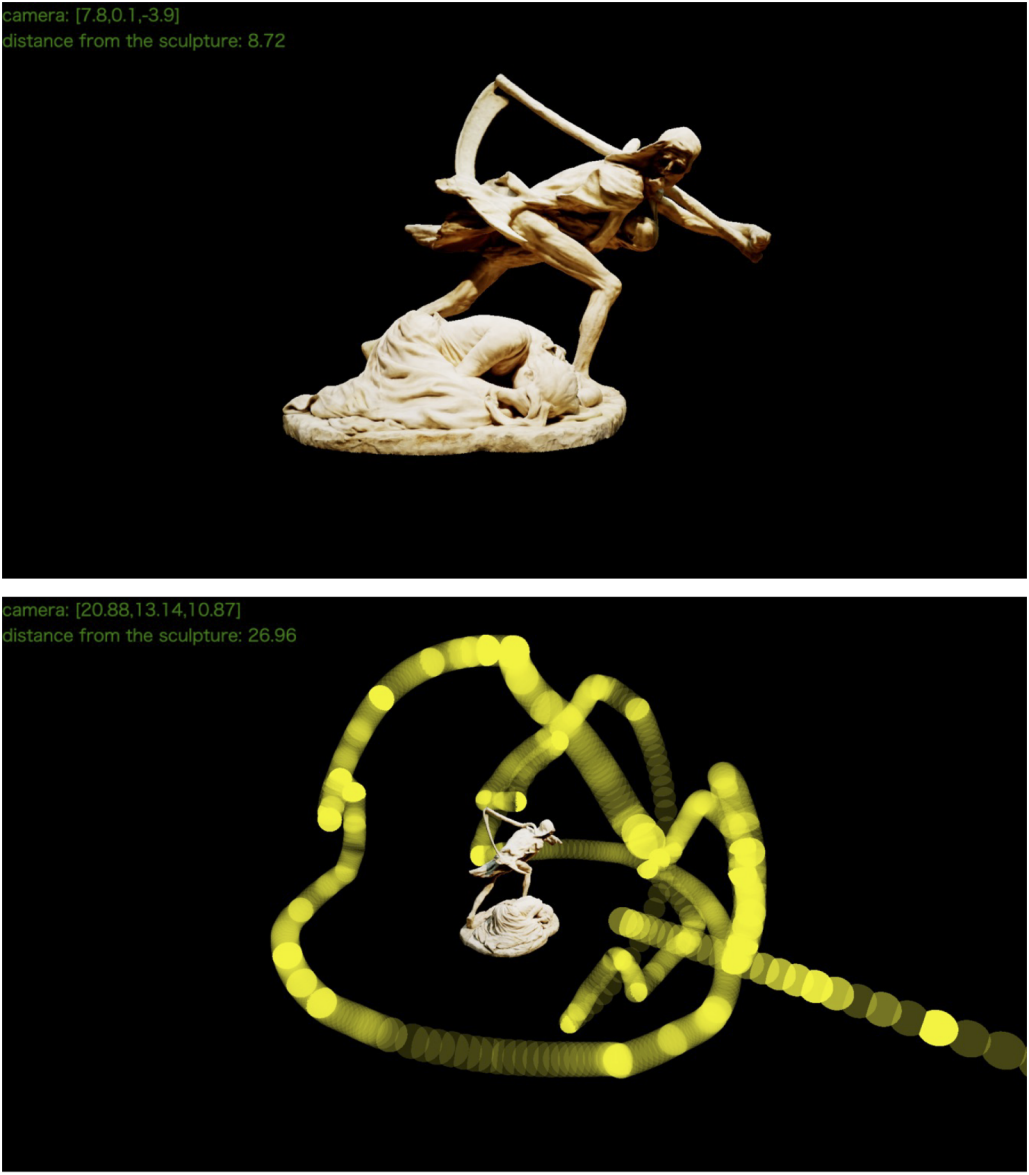
A 3D model of the sculptural work “Death and the Mother.” This model is used as a stimulus in the current experiment. Both images are created by the VESTA system. The first figure is seen from the default angle, with the camera in front of the work, reflecting what participants can see when starting the viewing phase. The second figure visualizes the locations of a participant's viewpoints during about 40 s, photographed from a position above and to the side. The translucent yellow sphere represents the position of the camera set by the participant at a certain point in time, with brighter yellow indicating that the camera stayed in that location for a longer duration. In this case, from the diagonal line extending downward to the bottom right, it can be observed that this participant approached to or retreated from the work in the front position. The denser concentration of yellow spheres in front of the artwork, with sparser distribution toward the rear, indicates that this participant mainly viewed the artwork from the front. The green text at the top left and yellow spheres are displayed for analysis and set up to be invisible to the participants during the experiment.

We utilized a 3D model of the sculptural work “Death and the Mother” created by Niels Hansen Jacobsen (1861–1941) as a stimulus (Figure 1). This model was downloaded from Sketchfab.com and displayed within the web application developed for this study.^2^ “Death and the Mother” is based on Hans Christian Andersen's fairytale “The Story of a Mother.” The narrative of this fairytale was used as part of the instructions presented to the participants, as described later.

This sculpture features three characters: “Death” (the Grim Reaper), a mother, and a child. Death is immediately noticeable due to the large scythe it carries, and the mother, who crouches at its feet, is also relatively easy to see. The child, being held against Death's chest, may be slightly difficult to see from the front, but it is likely to be visible when approached or viewed from different angles. Overall, although the artwork's composition can be roughly understood from the front, camera manipulation seems to encourage the examination of the details. Therefore, we believe that this artwork is suitable for observing voluntary behavior in front of it.

### Web Application: VESTA System

In the web application of this study (we named this Virtual Exhibition Space for Tracking and Analyzing [VESTA] system), participants had three types of interactions: (a) “Rotation,” (b) “Approach to/Retreat from Target,” and (c) “Shift of Target.” “Rotation” is the most basic action, and it refers to moving the camera's position on a spherical surface centered around the current target while keeping the gaze fixed on the target. In other words, in the “Rotation,” the camera orbits around the target to view the object's different sides hidden from view. While “Rotation” keeps the distance from the target constant, “Approach to/Retreat from Target” changes the distance while keeping the camera angle constant, similar to zooming in and out in a two-dimensional image. As the combination of “Rotation” and “Approach to/Retreat from Target” allows for viewing the entire artwork, “Shift of Target” is less frequently used. “Shift of Target” involves freely shifting the target coordinates that relates to the other two interactions, and it enables the camera to rotate around a point that is different from the original one. The participants were able to freely combine and manipulate these three types at any time.

The VESTA system recorded both the target coordinates that the participants were looking at and those of the camera's position with a time resolution of up to 60 frames per second (which may decrease depending on the performance of participants’ web-browsing environment). In other words, we accurately recorded the progression of each participant's operation of the VESTA system as numerical data without any omissions and immediately saved these data in TSV format on our cloud server. All functionalities of the VESTA system were implemented using Three.js, a JavaScript library based on WebGL (online demonstration: https://psychologykm.github.io/VESTA/demo/demo_recording_short.html).

### Experimental Conditions

We randomly assigned the participants to one of three conditions: the “Narrative Mode” group (*n* = 108); the “Process Mode” group (*n* = 110); and the “Creator Mode” group (*n* = 99). As the participants needed to complete complex experimental procedures online by themselves, some dropped out of the experimental procedure, which led to differences in sample sizes between conditions. However, as this would not significantly impact the analysis, neither did we replace or replenish the participants.

All conditions were manipulated solely by differences in the content of a single web page. Each page included a paragraph consisting of 10–12 sentences and a 2D photograph of the sculpture from which the stimulus was modeled.

In the “Narrative Mode” condition, participants were presented with a passage describing the plot of Andersen's “The Story of a Mother,” which had served as the inspiration for the sculpture. The text concludes by noting that the scene depicted in the sculpture corresponds to the final scene of the story, portraying the mother's realization of her child's death and her subsequent sorrow. Additionally, we provided participants with a partially visualized diagram illustrating the relationships between characters and motifs mentioned in the passage. In the “Process Mode” condition, we provided them with a passage explaining the typical creative process of a plaster sculpture, the category to which the current stimuli belong, along with a summarized diagram. In the “Creator Mode” condition, we attempted to induce participants to imagine themselves as sculptors creating a piece rather than providing information about the artwork. Before viewing the stimulus work, participants were asked to freely imagine ideas for a sculpture with the theme of “Death,” making their ideas clear as much as possible. Subsequently, they were shown images of the stimuli work, and then asked to refine their own imaginary creations with a focus on the differences from the presented stimuli work.

### Procedure

After reading a broad description of the tasks in an online document, each participant provided informed consent and began the tasks using their favored browser, accessing our web server. We required them to complete the tasks using a computer with a stable connection in a quiet, non-distracting environment.

On our website, participants first practiced operating the VESTA system by following instructions using a dummy 3D model. Once they felt that they had familiarized themselves with the operation, they proceeded to the aforementioned web page corresponding to the condition assigned to them (see Supplementary Table S1). We instructed them to read content related to the story if assigned to the “Narrative Mode” condition, read content related to the creative process if assigned to the “Process Mode” condition, or generate their own ideas for art-making if assigned to the “Creator Mode.” Participants were required to stay on the page for three minutes at least, enforced by the system. After this time elapsed and the participants judged that they had completed the page's contents, they proceeded to the VESTA system once again for the main trial. In this main trial, participants interacted with the stimulus for 100 s; shortening or extending this time period was not permitted. After the main trial phase, we asked the participants to freely report their thoughts in written form regarding their observations of the artwork (these data were not investigated further in this study) and answer items on a Likert scale. In the actual procedure, the last two phases—the main trial and reporting—were repeated for a purpose different from that of the current study. In the current analysis, we only use data collected the first time; thus, this repetition of the two phases has no relation to any of the results of this study.

### Measurements and Analysis

#### Likert Scale

The participants were asked to provide responses that reflected their various impressions of the stimulus. The dimensions measured were (a) liking, (b) admiration, (c) empathy, (d) imagination, (e) inspiration, (f) beauty, (g) boredom, (h) interest, and (i) nostalgia. We adopted (a)–(e) from previous studies (Matsumoto & Okada, 2021a, 2021b) on which the current study primarily relies theoretically. To enhance the comprehensiveness of the scale, (f)–(i) were adopted and translated from Hosoya (2020)'s study, which utilized multidimensional items of appropriate length. The only difference from the original scale was that we excluded one of the two items measuring (f) beauty from the current set of items as it used wording that could not be distinguished from (a) liking. Except for (e), which consists of five items (developed by Ishiguro and Okada [2015]), and (g)–(i), which consist of two items each, we measured all other dimensions using a single item. All items are shown in the Supplemental Table S2.

We conducted a MANOVA on all variables measured using Likert scales. Specifically, we treated the nine items comprising (a)–(i) as separate dependent variables and conducted a single test to examine whether the patterns of these variables differed across conditions.

#### Viewpoint Coordinates

We analyzed the coordinate information recorded by the VESTA system from two perspectives: the total traveled distance moved by each participant while viewing the stimulus, and the distribution of view-angles toward the artwork. The former analysis is relatively easy to understand; it involved calculating the Euclidean distance between all temporally adjacent pairs of the recorded 3D coordinates of the camera positions for each individual, summing these distances and log-transforming to obtain a score, denoted as “*d_total_*.” This score could be highly influenced by an individual's general tendency to move dynamically in 3D space. Therefore, we decided to calculate the same metric for the practice phase, which involved a dummy 3D model and was conducted before the experimental intervention, and include it as a covariate, denoted as “*d_cov_*,” in the difference testing model as analysis of covariance (ANCOVA).

The second analysis regarding view-angles required more complex modeling due to the circular structure of angle data, unlike typical variables that can be arranged on a one-dimensional number line, and the hierarchical structure in which the angle data at each time point is nested within individuals. To overcome this complexity, we adopted Bayesian estimation, which is well-suited for implementing complex hierarchical models due to its flexibility (Veenman et al., 2023).

**Figure 2.**
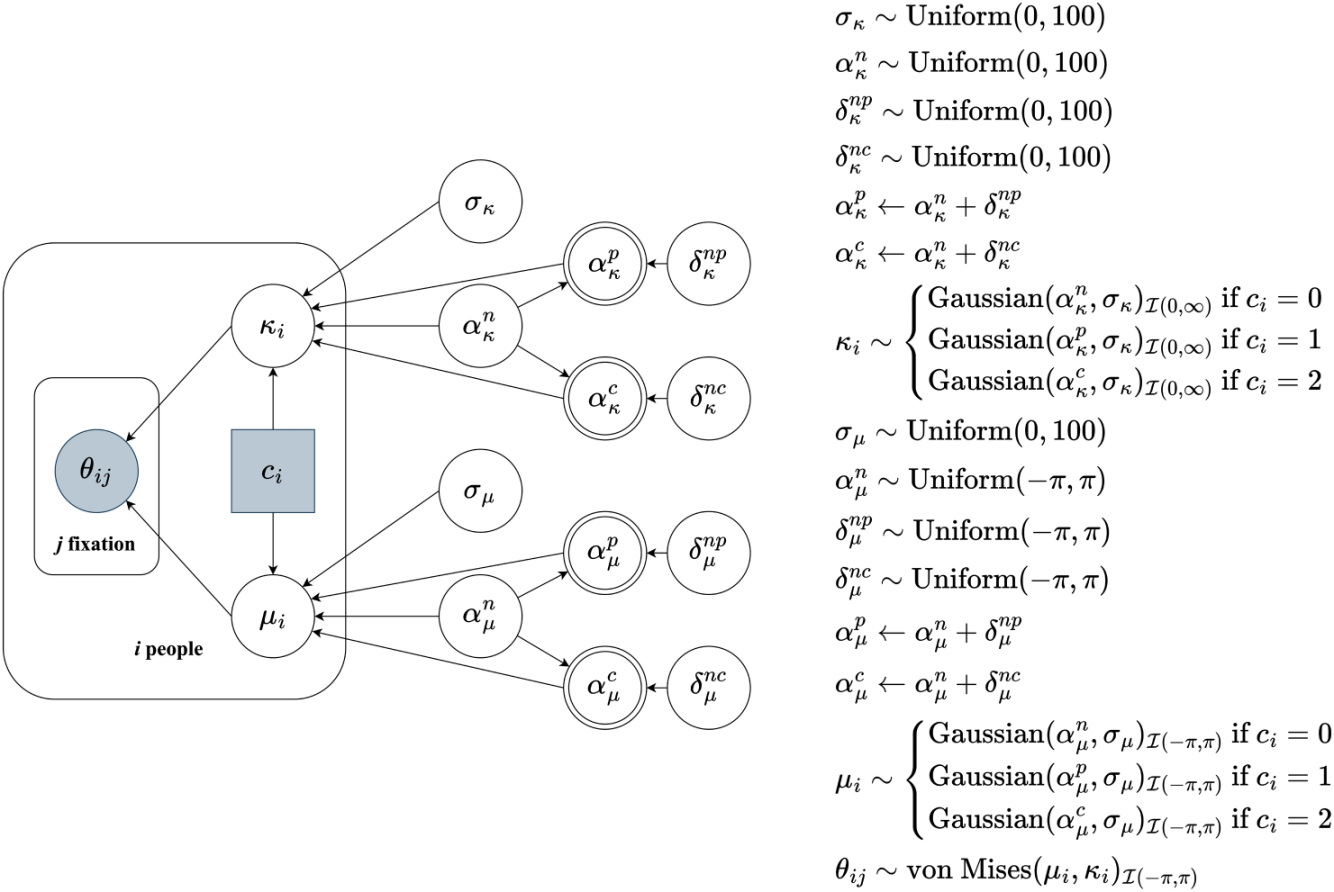
Graphical model for the hierarchical Bayesian analysis. Following Lee and Wagenmakers (2014, p. 18), each symbol carries the following meanings: circular/square nodes are used for continuous/discontinuous variables; shaded/unshaded nodes are used for observed/unobserved variables, and single-/double-bordered nodes are used for stochastic/deterministic variables.

Through Bayesian hierarchical analysis, we examined whether differences existed between conditions in individuals’ tendencies for view-angles. Figure 2 shows a graphical model for inferring this difference based on Lee and Wagenmakers (2014). The outcome variable is the view-angle at each time point.^3^ We denote the angle at the *j*th data point (or we can also call it *j*th “fixation,” as described below) of the *i*th participant as *θ_ij_*, which are modeled as draws from a von Mises distribution with mean direction *μ_i_* and concentration parameter *κ_i_*. The von Mises distribution is the circular analog to the Gaussian distribution. The *κ* in the von Mises distribution corresponds to the inverse of the *σ* parameter in the Gaussian distribution. When *κ *= 0, it is equivalent to a uniform distribution, and conversely, as *κ* increases, the probability of occurrence near the mean direction *μ* becomes higher. With the hierarchical approach, each participant is assumed to have a different von Mises distribution, the parameters of which further follow Gaussian distributions with parameters *α_κ_*, *σ_κ_*, *α_μ_*, and *σ_μ_*. The difference between conditions is considered only for the *α*s, means of Gaussian distributions, in the entire modeling: that is, if a participant is in the “Narrative Mode” condition (coded as *c_i _*= 0 in Figure 2), then *α *= *α^n^*; the “Process Mode” condition (*c_i _*= 1), *α *= *α^p^*; and in the “Creator Mode” condition (*c_i _*= 2), *α *= *α^c^*. *α^p^* and *α^c^* are given by *α^p ^*= *α^n ^*+ *δ^np^* and *α^c ^*= *α^c ^*+ *δ^nc^*, with *α^n^* and *δ*s representing the reference value and the differences from it, respectively; the larger the absolute value of *δ_μ_*, the further away each participant's mean view-angle in the given condition (e.g., the “Process Mode” condition) from those of the reference condition (the “Narrative Mode” condition). Conversely, a positive value of *δ_κ_* indicates that in that condition, participants tend to focus on a single location rather than various angles compared to the reference condition (if it is a negative value, the opposite holds true). Priors used for the estimated parameters *σ*, *α*, and *δ* are only weakly informative.

In addition to estimating each parameter, similar to hypothesis testing in frequentist statistics, we compared the H0 and H1 models. The H0 model is the null model where all *δ*s in the graphical model in Figure 2 are fixed to zero. In other words, it can be interpreted as one that completely disregards the differences between the three conditions. H1 imposes no constraints on the model. We calculated the Bayes Factor (BF_10_) to determine which model was preferable; a higher BF_10_ provides stronger support for the presence of differences between conditions. The analysis was conducted using Stan (Carpenter et al., 2017) and R package “bridgesampling” (Gronau et al., 2017).

While all the coordinate data for each individual were used to analyze the total traveled distance, for the Bayesian model regarding angles, only the camera coordinate data were included when they remained stationary at one location for 500 ms or more (such as fixations of eye movements). The primary reason for this was that in the model, all data collected at a high temporal resolution would result in a particularly high computational load. Second, and perhaps more importantly, we were interested in which angles participants actually continued to view the artwork from, rather than the countless points they passed through while searching for the desired view-angle. This is akin to analyzing the position of fixations rather than the saccadic trajectories in eye-tracking studies.

Among the 317 participants, those who met any one of the following criteria were excluded from the analysis of coordinates: those who moved the camera too far away from the target at any point during the observation (three participants), those who moved the camera an extremely short distance from the starting point (17 participants), or those whose number of recorded data points was especially low (averaging below 10 records/s) due to issues such as the performance of their personal computers (37 participants). Due to a single participant meeting multiple criteria, 56 individuals were excluded, leaving 261 participants eligible for the coordinate analysis (*n* = 84, 97, and 80 for “Narrative Mode” condition, “Process Mode” condition, “Creator Mode” condition, respectively).

## Results

All data and codes used in the analyses are available at https://github.com/psychologyKM/VESTA.

### Likert Scale

Supplemental Table S3 shows the means and SD of the participants’ scores on all Likert scales for each condition. We conducted a MANOVA to determine whether a significant difference existed in the pattern of those scores across conditions. Using Pillai's trace, the results revealed a significant main effect for the group of psychological measurements, *T *= 0.11, *F*(18, 614) = 2.01; *p *= .008; partial *η*^2 ^= .06, 95% CI [0.00, 0.07]. We conducted post-hoc comparisons using three separate MANOVAs to examine which pairs of the three conditions contributed to the above results. The analysis revealed significant differences between the “Narrative Mode” and “Process Mode” conditions, *T *= 0.10, *F*(9, 208) = 2.58; *p *= .008; partial *η*^2 ^= .10, [0.01, 0.15], and between the “Narrative Mode” and “Creator Mode” conditions, *T *= 0.10, *F*(9, 197) = 2.30; *p *= .018; partial *η*^2 ^= .10, [0.00, 0.14], while no significant difference existed between the “Process Mode” and “Creator Mode” conditions, *T *= 0.06, *F*(9, 199) = 1.35; *p *= .214.

Additionally, we conducted separate ANOVAs for each variable to perform post-hoc tests comparing the variances between the “Narrative Mode” and “Process Mode” conditions (denoted as “N-P contrast”) and between the “Narrative Mode” and “Creator Mode” conditions (denoted as “N-C contrast”) as both pairs had showed significant results in the post-hoc MANOVAs. In N-P contrast, ANOVAs revealed only two significant differences: (c) empathy, *F*(1, 216) = 11.93; *p *< .001; *η*^2 ^= .05, 95% CI [0.01, 0.12], and (d) imagination, *F*(1, 216) = 4.22; *p *= .041; *η*^2 ^= .02, [0.00, 0.07]. For both variables, the “Process Mode” condition showed higher mean value than the “Narrative Mode” condition. In N-C contrast, ANOVAs revealed only two significant differences: (c) empathy, *F*(1, 205) = 6.31; *p *= .013; *η*^2 ^= .03, [0.00, 0.09], and (h) interest, *F*(1, 205) = 4.59; *p *= .033; *η*^2 ^= .02, [0.00, 0.08]. For both variables, the “Creator Mode” condition showed a higher mean value that the “Narrative Mode” condition. These results were obtained from exploratory post-hoc tests, and it is difficult to draw definitive conclusions from them as, except for (c) empathy, they could be insignificant after correction for multiple comparisons, such as Bonferroni adjustment. Moreover, if we use Bonferroni adjustment for post-hoc MANOVAs, even the entire difference between the “Narrative Mode” and “Creator Mode” conditions could be insignificant. Bearing these limitations in mind, we attempt to interpret these results in the discussion section.

### Viewpoint Coordinates

#### Total Traveled Distance

The mean and SD of *d_total_* for each condition were 5.77 (0.49), 5.86 (0.44), and 5.68 (0.59), for the “Narrative Mode,” “Process Mode,” and “Creator Mode” condition, respectively. We conducted an ANCOVA test to determine whether a significant difference in *d_total_* existed across conditions. The ANCOVA result revealed a significant main effect for *d_total_*, *F*(2, 257) = 3.47; *p *= .032; partial *η*^2 ^= .03, 95% CI [0.00, 0.07], with a significant covariate effect, *F*(1, 257) = 14.34; *p *< .001; partial *η*^2 ^= .05, [0.01, 0.11]. Post-hoc comparisons were conducted using three separate ANCOVAs to examine which pairs of the three conditions contributed to the above result. The analysis revealed a significant main effect only for the comparison between the “Process Mode” and “Creator Mode” conditions, *F*(1, 174) = 7.04; *p *= .009; partial *η*^2 ^= .04, [0.00, 0.11], and insignificant main effects between the “Narrative Mode” and “Creator Mode” conditions, *F*(1, 161) = 1.04; *p *= .309), and between the “Narrative Mode” and “Process Mode” conditions, *F*(1, 178) = 2.59; *p *= .109). The significance of these results did not change whether Bonferroni correction was applied.

#### Distribution of View-Angles

**Figure 3.**
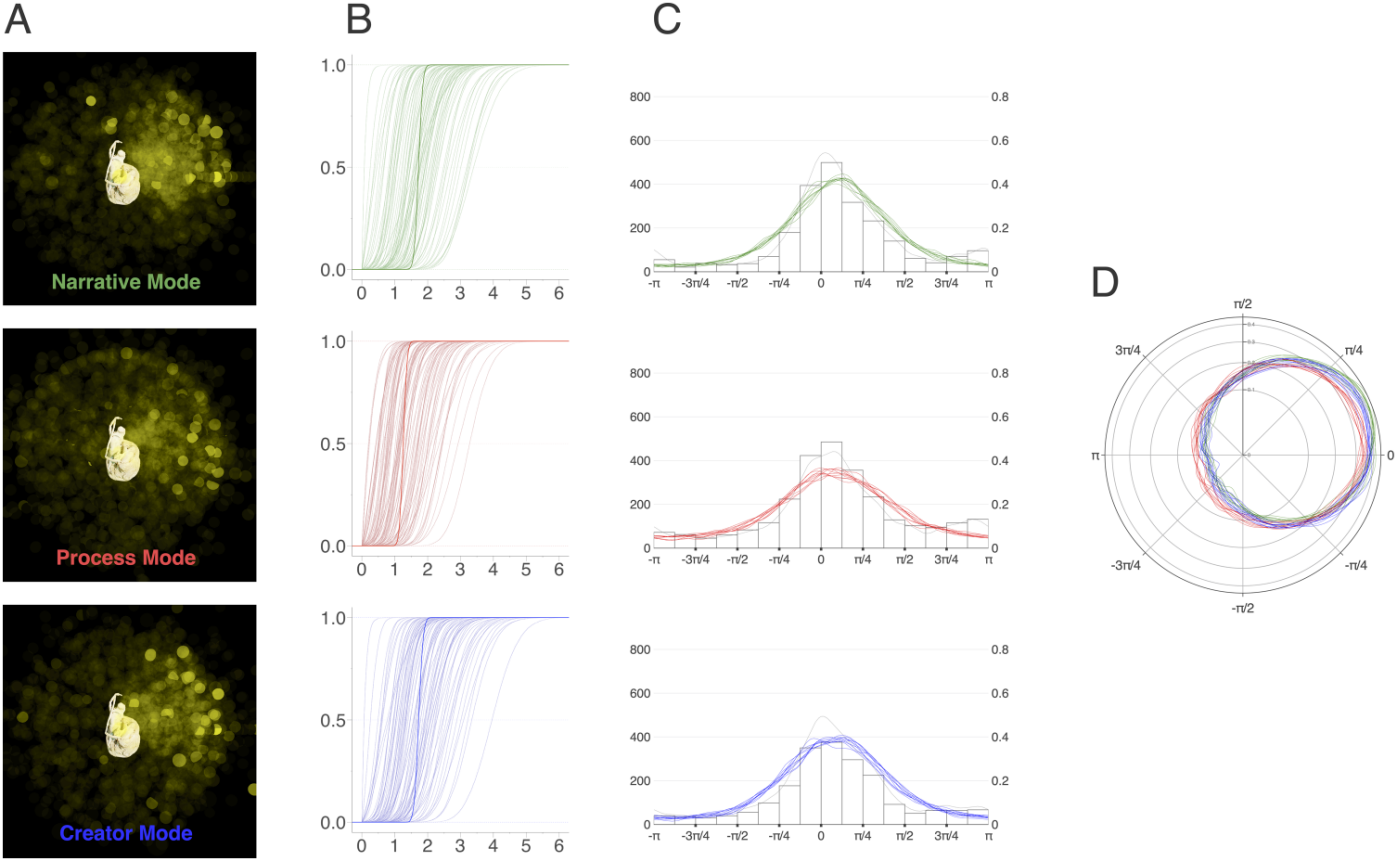
Recorded data of viewpoint coordinates and estimations of parameters. (A) Images captured from above in the VESTA system, overlaying the stimulus and the recorded coordinates of the cameras distributed around it for each condition. To clearly illustrate the trends, the plotted data points are randomly sampled from the entire dataset at a 1% probability, and the transparency of the yellow spheres indicating camera positions is set higher than in Figure 1. (B) Plots of empirical cumulative distribution function curves for Markov Chain Monte Carlo (MCMC) results of *α_κ_*s and *κ_i_*s for each condition. Within each plot, the thick and bold line represents *α_κ_* (group-level parameter), while the thin and faint lines represent *κ_i_* (individual-level parameter). Consistent with the results in Table 1, the “Process Mode” condition shows that the distributions of *α_κ_* and *κ_i_*s are generally concentrated at lower values compared to the other two conditions. (C) Plots of estimated probability density function curves overlayed on the actual data distribution. Within each plot, the gray histogram shows actual *θ_ij_*s’ distribution (with gray curve of kernel estimated density). Colored lines show the curves of kernel estimated density of post predictive distributions of *θ_ij_* (randomly sampled from MCMC results ten times for each condition). The presence of a density peak near 0 indicates that fixations were concentrated toward the front of the stimulus in all conditions. Among all conditions, only the peaks of the red lines in the “Process Mode” condition do not reach 0.4. This means that MCMC results reflected the difference in the degree of concentration of fixations between conditions in the posterior predictive distribution. (D) An integrated plot of every colored line in Figure 3C, with them redrawn onto polar coordinates. The direction of these polar coordinates aligns with the orientation of space in Figure 3A. To eliminate area distortion, the radius scale is taking the square root. The slight leftward deviation of the red circle from the other circles indicates a tendency similar to that observed in Figure 3A, where, in the “Process Mode” condition, fixations tend to concentrate toward the rear of the stimulus compared to the other conditions.

**Figure 4.**
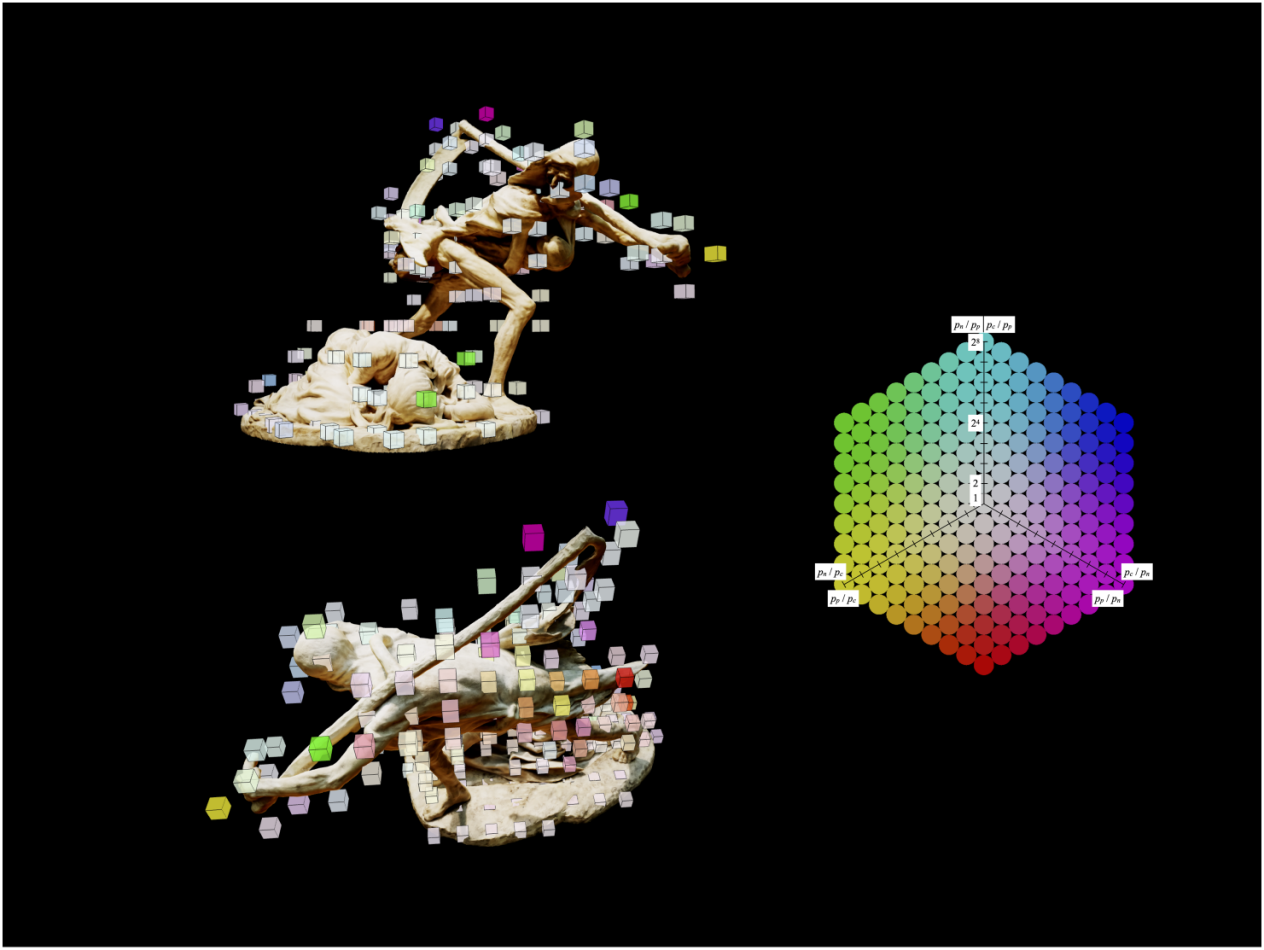
Images captured in the VESTA system, overlaying the stimulus and the cubes representing the differences in the total time observed at each area of the stimulus between conditions (the top left image was taken from a diagonal front perspective and the bottom left one from an upper rear perspective). Each cube is colored based on the following rule: green for more gazes in the “Narrative Mode” condition, red for more gazes in the “Process Mode” condition, and blue for more gazes in the “Creator Mode” condition. The intensity of each color component increases proportionally to the ratio of gazes. For example, a light green cube near the crouching mother's head means that participants in the “Narrative Mode” condition tend to spend much more time looking at that part of the stimuli (more precisely, keeping it within the viewing frame). The average proportion of time participants spent on each area for each condition was calculated as *p_n_*, *p_p_*, and *p_c_* (respectively denoting Narrative, Process, and Creator). The color was determined based on the ratio of these three values, as shown in the color chart on the right. As the differences between all conditions decrease, the color approaches white. Cubes that are closer to white than a certain threshold are removed from the figure for clarity. This figure was created based on the calculation of intersections between lines and 3D objects using the THREE.raycaster class in the Three.js library.

Using the VESTA system, we plotted the coordinates of camera positions adopted by the participants for each condition, overlaying them on the stimulus (Figure 3A). In other words, these images visualized the participants’ “viewpoints” while viewing the artwork. Intuitively, this figure suggests that while in the “Narrative Mode” and “Creator Mode” conditions, the camera's position seemed to show a strong concentration toward the front of the sculpture (rightward in the images); in the “Process Mode” condition, by contrast, the tendency toward the front became more modest, and the frequency of camera movement toward the rear of the sculpture was relatively higher (leftward in the image). In addition, we plotted the areas where participants spent more time viewing (or more precisely capturing on camera) the surface of the stimulus (Figure 4). While Figure 3A is about “from where they viewed,” Figure 4 shows “where they looked at.” As we did not have any data on the participants’ eye movements, our estimation of the amount of time participants spent looking at each area remains approximate. Nevertheless, this visualized result suggests that while participants in the “Narrative Mode” and “Creator Mode” conditions directed their cameras more toward the characters’ faces, participants in the “Process Mode” condition tended to move their cameras round to the artwork's rear.

**Table 1.** Posterior summaries of Markov Chain Monte Carlo results for the H0 and H1 model.

Model	Parameter	2.5%	*M*	97.5%	*SD*
H0	$\sigma_{\kappa }$	0.747	0.847	0.962	0.055
	$\alpha_{\kappa }$	1.430	1.548	1.670	0.061
	$\sigma_{\mu }$	0.336	0.384	0.440	0.026
	$\alpha_{\mu }$	0.248	0.307	0.365	0.030
H1	$\sigma_{\kappa }$	0.721	0.820	0.934	0.055
	$\alpha_{\kappa }^{n}$	1.519	1.724	1.929	0.105
	$\delta_{\kappa }^{np}$	−0.739	−0.466	−0.195	0.139
	$\delta_{\kappa }^{nc}$	−0.287	0.003	0.300	0.149
	$\sigma_{\mu }$	0.336	0.385	0.441	0.027
	$\alpha_{\mu }^{n}$	0.246	0.344	0.444	0.050
	$\delta_{\mu }^{np}$	−0.181	−0.042	0.100	0.072
	$\delta_{\mu }^{nc}$	−0.216	−0.074	0.066	0.073

In the Bayesian estimation of the H0 (without any assumption of differences across conditions) and H1 (assuming differences across conditions) models, the convergence of the Markov chain Monte Carlo chains was validated by *R-hat* statistics (all *R-hat* values were less than 1.01). Table 1 presents the estimated group-level parameters (for details of Markov Chain Monte Carlo [MCMC] procedures, see Supplemental Table S4). The BF_10_ calculated for 10 iterations ranged from 88.488 to 4189.818, indicating that the H1 model is better at explaining the data compared to the H0 model (this follows the recommendation from Veenman et al. (2023) to conduct stability analysis when using bridge sampling, and these 10 iterations—the process including both MCMC and bridge sampling—were run solely for BF calculation). Considering that the model comparison favored the H1 model and that the estimated parameter *α_κ_* (indicating the concentration of angles in the von Mises distribution) was relatively lower in the “Process Mode” condition, it became apparent from the data that each participant in the “Process Mode” condition viewed the stimulus from a wider range of angles (Figure 3B, 3C, and 3D).

## Discussion

Our experiment aimed to capture the changes in virtual art-viewing processes elicited by different contexts. The main results are two. First, the psychometric analysis (MANOVA) shows that the three groups of participants had different patterns of responses to a group of questions about affective and cognitive states. Second, the coordinate analyses (ANCOVA for total traveled distance and hierarchical Bayesian analysis of directional data) show that the three groups of participants had different patterns of viewpoint movement in a virtual space. These main results support our hypotheses, proving the emergence of distinct “modes” of viewing for each condition in the current experimental design.

As for psychometric analysis, we also found implications from the explorative post-hoc analyses. Participants in the “Process Mode” condition exhibited higher empathy toward the sculptor and more expanded imagination from the artwork than those in the “Narrative Mode” condition. These results align with Matsumoto and Okada's (2021b) results, where a focus on the creative process significantly facilitated empathy, while imagination increased only with a significant trend. In contrast, participants in the “Creator Mode” condition exhibited higher empathy and interest than those in the “Narrative Mode” condition. Due to the lack of similar previous studies in the “Creator Mode” condition and the inability to definitively determine significance from a multiple comparison perspective, the latter comparison is more challenging to interpret. Nevertheless, these results seem to partially reflect the differentiation in the participants’ impressions of the artwork induced by the current experimental intervention for three conditions. Interestingly, we found no significant effects for liking and admiration, which were expected to be facilitated by focusing on the creative process. This might be because the “Narrative Mode” and “Creator Mode” conditions, which were compared to the “Process Mode” condition, had their own effects in promoting liking and admiration for the artwork, unlike the contrast group in previous studies (Matsumoto & Okada, 2021a, 2021b).

Regarding the coordinate analyses, we emphasize that they offer information about changes within each condition that could not be addressed through psychometric analyses. Based on the analysis of viewpoints’ traveled distance, participants in the “Process Mode” condition moved their perspectives over longer distances within the virtual space, especially compared to the participants in the “Creator Mode” condition. Furthermore, according to the directional analysis, participants in the “Process Mode” condition tended to approach the artwork from a broader perspective rather than focusing on it from a single direction. These characteristics of participants’ moving patterns in the “Process Mode” condition suggest that they explored the surroundings of the presented artwork more extensively, fueled by a unique interest sparked by the experimental intervention.

Why did participants in the “Process Mode” condition tend to explore a virtual space more widely? When compared to those in the “Narrative Mode” condition, this can be easily interpreted. In many cases, informative locations for viewers focusing on a narrative aspect in visual art, such as characters’ facial expression, do not disperse throughout a work but converge in a few areas (although we could have an exceptional setting, such as viewing Gothic sculptures featuring numerous figures). Our setting of the “Narrative Mode” condition is no exception as the priming text was likely to lead viewers to pay attention to the story behind the scenes and they could perceive the relationships or expressions of each character by looking at the work directly from the front. Accordingly, participants in the “Narrative Mode” condition spontaneously stood in front of the artwork and remained there, continuing to observe. In contrast, in the “Process Mode” condition, participants were likely to pay attention to parts of the artwork that evoked its creative process in their minds. Those parts, such as surface texture or unevenness, thickness, curvature, and overall arrangement, are ubiquitously found throughout the work. Therefore, in the current setting, it seems natural that viewers under a “process mode” are motivated to explore a wider space to collect dispersed information.

Conversely, when comparing “Creator Mode” and “Process Mode,” the difference is more difficult to interpret. Participants in the “Creator Mode” could have yielded results closer to those in the “Process Mode” as both conditions seemed to provide the creator's perspective, but it did not happen. One possible reason is that participants in the “Creator Mode” condition might have been focusing on a narrative aspect of artwork as a “dramatist” rather than as a “sculptor.” If so, participants in the “Creator Mode” condition might not have been interested in what kind of physical creative process was behind the sculpture but might have been more interested in the narrative or mental aspect of the creative process, such as how unique the story of the scene was or how it can be further modified (for segmenting the aspects of the process of creation, see Matsumoto and Okada (2021a)). Thus, the perspective brought about by the intervention in the “Creator Mode” condition can be closer to that in the “Narrative Mode” than to that of the “Process Mode.” In addition, the physical aspect of creating plaster sculptures is rarely known and thus attracts less attention without being provided with specific knowledge. Considering that novices tend to pay attention to semantic content rather than formal elements (Bullot & Reber, 2013; Cupchik, 1992; Cupchik & Gebotys, 1988; Parsons, 1987; Schmidt et al., 1989), it seems highly plausible that the participants in the “Creator Mode” condition were not led into focusing on the formal features of the artwork related to the physical aspect of the creative process. Furthermore, they might have failed to mimic the thought process of actual creators due to the lack of the knowledge about the sculpture, even though the instruction was to “think as a creator.” This is one of the limitations of this study, and addressing this issue would likely require designing longer and more systematic interventions or recruiting experts with knowledge of creation as participants.

An important aspect to note here is that the differences between conditions emerged without explicit instructions such as “how to view the work.” Differences in psychological and behavioral indicators can arise due to the instructed viewing methods (e.g., Cotter et al., 2023; Matsumoto & Okada, 2021b). In contrast, in this study, particularly in the “Narrative Mode” and “Process Mode” conditions, participants were only presented with prior information related to the artwork. The results suggest that the participants in this experiment spontaneously altered their modes of viewing based on their intrinsic interests. Furthermore, considering that changes in modes of viewing can arise depending on nuanced contextual differences, it is predicted that such changes might occur frequently in daily life.

It should also be noted that the differences between conditions observed in this study are not very large, as can be seen from the plots in Figure 3. This is likely attributable to the unobtrusive nature of the instructions used. Since the primary focus of this research is not to identify interventions that produce large effects, but rather to demonstrate the effectiveness of the methodology, the small effect sizes successfully highlight the high sensitivity of this approach.

### Concept of Mode

This study focused on the concept of “mode” of viewing, which can effectively explain how an individual's art experience varies depending on the situation. Moreover, it is suitable for capturing changes that encompass multiple aspects (positive or negative) rather than a single-dimensional change. Efforts to examine esthetic experiences through multidimensional measures, which have emerged in recent years (e.g., Hosoya, 2020; Kenett et al., 2023; Nummenmaa & Hari, 2023; Specker et al., 2021), may also provide further benefit by associating individual multidimensional patterns with different modes. Researchers could also consider incorporating physical indicators into this measure, as in this study. One example of the “mode” and its accompanying changes in multiple indicators can be described as follows: when individuals enter a “mode” focusing on the artwork's process (as long as the viewing situation is akin to the current one), they will be engaged in physically extensive exploration and have a specific structure of mental state, such as high empathy toward the artist. Systematically exploring how variables are causally related to each other within a single mode and identifying other conceivable modes remains a challenge for future research.

It is important to note that these modes may be more constant than those assumed in this study. Although each condition was associated with a single dominant mode in this experiment, at the microlevel, the modes may shift incessantly throughout the viewing period. For example, participants in the “Process Mode” condition may not always be focused on the process of creating the artwork; in some moments, their attention is likely to shift to considering what scene the sculpture represents in a story. Future research is required to elucidate the temporal dynamics of the transition of one mode into another.

Another point to note is that we can conceive of different levels of “modes.” The terms “Narrative Mode” and “Process Mode” refer to what individuals focus on, whereas the term “Creator Mode” refers to who is viewing or what kind of (or in what state) person is viewing. Two modes defined at different levels refer to the same phenomenon or conceptually overlap despite their apparent difference. Actual creators, when entering the “Creator Mode” to view others’ works, may process the artwork from multiple perspectives, potentially switching between the Narrative Mode, Process Mode, or even other modes. Therefore, it is necessary to consider the hierarchical relationship of the modes in future research.

### Scope of the Methodology

In this study, we implemented a novel methodology for investigating art-viewing behavior in virtual spaces, which are characterized by two elements: (a) measuring and analyzing viewers’ movements and (b) utilizing non-immersive VR. As a result of this methodology, we were able to capture changes in the participants’ modes of viewing, which were clearer and more informative than what could be inferred from psychological indicators alone. Therefore, the efficacy of this methodology was sufficiently demonstrated.

Based on our methodology, we could envision further research endeavors. For example, as non-immersive VR allows numerous participants to participate in an experiment, researchers may use machine learning to accurately predict psychological states (such as interest or boredom) or subsequent behavioral patterns (such as when to stop engaging with the artwork) from movement indicators. Furthermore, it can even be applied to collective viewing behaviors in social groups of two or more individuals. Beyond the framework of scientific research, we believe that it has practical applications in real-world settings. In particular, as an increasing number of virtual viewing situations are created by museums and tourism service providers, a detailed analysis of the individual viewing process using the VESTA system or something similar could be of value both commercially and educationally. In the big picture, the scope of this methodology is not limited to typical art appreciation, but it may also have other applications. Cognitive scientists interested in any cognitive process related to active searching can easily incorporate this methodology into their experiments. If we consider an educational setting, we can gather valuable insights into each learner's attention or identify which parts of a displayed object are overlooked by the majority. Other kinds of virtual spaces such as the metaverse which are already widely used as services would also be included within the scope of this methodology.

Although this study primarily focused on the art-viewing in virtual spaces, the insights gained from this methodology are expected to contribute to examining esthetic experiences in the real world. This expectation is corroborated by the finding that people's reaction in the “Process Mode” condition of the present experiment closely aligns with theoretical predictions from prior research conducted in real-world settings. Furthermore, as already noted in the introduction, it is worth highlighting that non-immersive VR closely resembles the conventional viewing conditions conducted in traditional research on 2D monitors, as opposed to immersive VR. Nonetheless, it is essential to proceed with caution and carefully verify the extent to which generalizations are possible in future studies. In particular, a significant distinction ought to be made with regard to bodily sensations between walking around an artwork and rotating the camera. This underscores the importance of examining the gaps between virtual and real bodily perceptions.

We should also note that the current methodology makes differences in movement clearer in some cases; conversely, it lets differences in psychological indicators likely emerge in other cases. This will depend on the type of artwork and mode of viewing. To overcome this issue, it is important to conduct a multifaceted assessment that integrates psychological measures and movement, as has been done in this study.

### Limitations

This study has two main limitations. First, it focuses on experiences with artworks in non-immersive VR, which differ from experiences with the real environments, their 2D representations, or immersive VR environments. Specifically, using virtual spaces enables movements that are difficult to achieve in reality, such as viewing large artworks directly from above or below. This likely leads to differences in both movement trajectories and psychological experiences. Moreover, while immersive VR can cause discomfort when moving quickly through space, similar to real-life experiences, non-immersive VR is less likely to produce such effects. This could result in differences in the speed and range of exploratory behaviors between them. Therefore, the differences in exhibition formats should not be overlooked, and this study did not empirically examine these distinctions. Researchers should be careful not to overgeneralize the findings derived from the current methodology.

Second, the geometric analysis used in this study is just one of the potentially numerous ways to process the data. For example, since the viewpoint coordinates are recorded with time information, a time-series model could also be applied. In future research based on the current methodology, its authors may need to develop analysis methods tailored to their specific topics for themselves. While the richness of the data allows for flexible analysis, it also imposes a burden on researchers to determine the appropriate analytical approach. In the future, a systematic and comprehensive overview of possible analyses for data obtained through the current methodology will be necessary.

### Conclusion

Through the investigation of changes in people's “modes” of viewing, we successfully demonstrated the effectiveness of integrating measurements of movement with non-immersive VR in examining art-viewing experiences. This methodology not only provides insights into virtual viewing experiences, but it is also likely to offer valuable implications for real-world viewing experiences in a more efficient way. In particular, analyzing how the physical “viewpoint” is positioned in space provides a powerful means to infer what kind of mental “viewpoint,” or what kind of “mode” of viewing, people have in their minds. With the current methodology being both applicable for different areas and sharp for specific research questions, this study paves the way for future research.

## Supplemental Material

sj-docx-1-ipe-10.1177_20416695251314182 - Supplemental material for Visualizing the change of “viewpoints” in 3D virtual art exhibition Supplemental material, sj-docx-1-ipe-10.1177_20416695251314182 for Visualizing the change of “viewpoints” in 3D virtual art exhibition by Kazuki Matsumoto and Takeshi Okada in i-Perception

## Notes

1. Some previous studies have used nonimmersive VR in the context of art. Most have been conducted as visitor studies in virtual museums or virtual galleries and have succeeded in demonstrating the usefulness of the method (e.g., Cotter et al., 2023; Sundar et al., 2015; Sylaiou et al., 2017). Among them, an exceptional study analyzed movement data while viewing artworks (Rodriguez-Boerwinkle, 2023), as proposed in the current study. Our methodology could be seen as providing theoretical grounding with new empirical data to this pioneering practice. From another point of view, Rodriguez-Boerwinkle (2023) focused on implementing a gallery-like space where multiple artworks were exhibited and which was closer to the virtual spaces actually in operation, such as the museums’ websites. In contrast, the current study conducted experiments focusing on participants’ responses to a single artwork as traditional empirical aesthetic studies have done, primarily exploring the way to extend existing methodologies adopted by psychologists.

2. This work is based on “The Death and the Mother” (https://sketchfab.com/3d-models/the-death-and-the-mother-793a94b673334484b1bcfbd20c8bae19) by Geoffrey Marchal (https://sketchfab.com/geoffreymarchal), licensed under CC-BY-NC-4.0 (http://creativecommons.org/licenses/by-nc/4.0/).

3. In this study, we calculated the view-angle θ (−π ≦ θ < π) using two-dimensional coordinates on a plane parallel to the ground where the stimulus was placed, with the origin [0, 0] (the center of the stimulus) as the reference point. The default angle set when entering the VESTA system page, where the camera is directly in front of the sculpture (as the above image in Figure 1), was defined as 0. From this starting position, rotating the camera counterclockwise by 90° around the artwork's center (viewed from above) corresponds to θ = 1/2π, and rotating 90° in the opposite direction corresponds to θ = −1/2π.
