# Supplementary material for: Visualizing the change of “viewpoints” in 3D virtual art exhibition
Source: Iperception. 2025 Jan 31;16(1):20416695251314182. doi: 10.1177/20416695251314182 (PMC11786277; doi:10.1177/20416695251314182)
Supplement: sj-docx-1-ipe-10.1177_20416695251314182 - Supplemental material for Visualizing the change of “viewpoints” in 3D virtual art exhibition [file sj-docx-1-ipe-10.1177_20416695251314182.docx]

Supplementary Material

Table S1
*Instructions presented in each condition.*

| Variable | “Narrative mode” condition |
| --- | --- |
| Common text  (inserted with a 2D photograph of the sculpture at {common text} in each condition) | 右の写真は、デンマークの作家・ Niels Hansen Jacobsen（1861-1941）の「死と母」という彫刻作品である。この作品は、鎌を持った死神がある母親の子供を連れ去っていくという場面を描いたもので、母親が子供と死別した悲しみが表現されている。 彫刻の大きさは、幅、奥行き、高さがそれぞれ130cmから200cmと、ほとんど等身大でできた作品である。その素材は石膏でできている。 |
| “Narrative mode” condition | {common text} この作品の場面は、「ある母親の物語」と題されたアンデルセンの創作童話のストーリーが元になっている。そのあらすじとは、以下のようなものである。 「一人の母親と病気で今にも死にそうな子どもがいるところに、死神が現れ、その子どもを連れ去ってしまった。子どもを失いたくない母親はあらゆる手段を尽くして死神を追いかけ、体のあちこちが傷付いた状態になりながらも、遂には死神の住みかまでたどり着いた。死神の住みかには人間たち一人ひとりの寿命を示す木や花が植えてあった。その木や花が枯れるとそれに対応する人は死ぬという仕組みになっていた。母親は、そこにいたある老婆の勧めに従って、自分の子どもの花を死神が抜くのなら、他の花も抜くと死神を脅した。しかし死神は、関係のない花を抜くことは、自分と同じような不幸を他の親にも与えることになると逆に母親を諭した。そして、死神は井戸の水に幻の景色を映し出し、母親の子どもと、母親が抜こうとした花の子どもの2人が死ななかった場合に未来にどのような姿になるかを母親に見せた。そして母親は、自分の子どもを神の国に連れて行って欲しいと死神に言い、死神は子どもを神の国に連れて行ってしまった（子どもは死んでしまった）。」 この彫刻の場面は物語の最後の場面に相当し、もはや死神を追いかけることなく自分の子どもの死を認めてはいるものの悲しみに暮れ続ける母親の様子が映し出されている。 |
| “Process mode” condition | {common text} 一般的に、石膏の彫刻作品は、以下のような過程で制作される。 石膏の彫刻を作るためには、まず粘土で同じ形の像を作る必要がある（塑像[そぞう]と呼ばれる）。粘土は加工しやすい代わりに柔らかく、自重でも崩れたり変形したりしてしまうため、形が崩れないように、針金や木片を使って「心棒」と呼ばれる中心部の支えとなる構造を先に作り、その周りに粘土を塗りつけるように形を作っていく（鉄筋コンクリートで鉄筋を芯にしてコンクリートを補強するように、心棒を芯にして粘土を補強する）。粘土をヘラなどを使いながら形を整える際には、目や鼻などの各パーツの位置が少しずれるだけでも印象が大きく変わってしまうため、それらが歪まずに正しい位置に来ているかを確認しながらフォルムを調整する。 粘土による塑像が完成したら、その周囲全体を覆う形で液状の石膏を振りかける。この石膏が固まってから、あらかじめ入れておいた線に沿って石膏を分割して取り外すと、中が塑像の形に空洞になった石膏の「型」ができ上がる。ただし、この型も空洞状で構造は脆いため、実際には針金の補強などが入る。この空洞の型の中に改めて液状の石膏を流し込み（その際、型の石膏と流し込んだ石膏が融合してしまわないように、型の方にだけ石鹸水を染み込ませるなどする）、固まるのを待ってから外側の型だけを外せば、粘土の塑像と同じ形の石膏の彫刻作品ができあがる。 |
| “Creator mode” condition | ここで、次のことを想像してみてください。 あなたがもし、自由に形を整えて彫刻を作れるとして、この作品とは別の形で「死神」をテーマにした作品を作るとしたら、どのような彫刻作品を作りますか？ なるべく細かい部分まで想像を働かせて、考えてみてください。 ただし、物理的に可能な範囲の形を考えるようにしてください（例えば、宙に浮いているようなものはここでは作れないものとします）。 その作品のアイデアがある程度固まったら、「自分の作品を洗練させるために他の人の作品を見る」というつもりで、次の作品と説明文を見て、「自分のアイデアと何が似ていて何が違うか」などの観点からしばらく考えてみてください。 {common text} ※ここで色々なことを考えていただいたのは作品に対して普段と違う見方をするためのメソッドであり、いい作品のアイデアが思いつかなかったとしても、問題ありません。 ※特に考えたいことが無くなったら、次に進んでいただいて構いません。 |

Table S2
*Scales adopted in the experiment.*

| Variable | Text | Answer options |
| --- | --- | --- |
| Liking | I like this work.  この作品が好きだ | 7-point scale from “Agree very strongly” (6) to “Disagree very strongly” (0) |
| Admiration | I felt admiration for this work.  この作品に感嘆した |  |
| Empathy | I emphasized with the creator of this work.  この作品の作者に共感した |  |
| Imagination | My imagination expanded from the work.  この作品から想像が広がった |  |
| Inspiration | 1. I felt inspired.   インスピレーション（触発）を感じた |  |
|  | 1. New images and ideas came to mind.   新しいイメージやアイディアが湧いた |  |
|  | 1. I was excited.   わくわくした |  |
|  | 1. I wanted to express myself in some way.   自分も何か表現したくなった |  |
|  | 1. I wanted to actually do something.   実際に何かをしてみたくなった |  |
| Beauty | I find this work beautiful.  この作品は美しいと思う |  |
| Boredom | 1. This work bores me.   この作品は退屈だ |  |
|  | 1. I feel indifferent to this work.   この作品に特別なものを感じない |  |
| Interest | 1. This work makes me curious.   この作品が気になる |  |
|  | 1. This work sparks my interest.   この作品に興味が湧く |  |
| Nostalgia | 1. This work makes me feel nostalgic.   この作品は自分に郷愁を感じさせる |  |
|  | 1. This work makes me feel sentimental.   この作品は自分を感傷的にさせる |  |

| Variable | “Narrative mode” condition | “Process mode” condition | “Creator mode” condition |
| --- | --- | --- | --- |
| Liking | 2.64 (1.36) | 2.64 (1.13) | 2.82 (1.45) |
| Admiration | 2.11 (1.06) | 2.22 (1.14) | 2.26 (1.19) |
| Empathy | 2.40 (1.16) | 2.95 (1.18) | 2.84 (1.36) |
| Imagination | 1.67 (1.03) | 1.96 (1.10) | 1.86 (1.13) |
| Inspiration (1) | 2.43 (1.37) | 2.45 (1.14) | 2.25 (1.26) |
| Inspiration (2) | 3.12 (1.29) | 3.22 (1.12) | 2.85 (1.34) |
| Inspiration (3) | 3.44 (1.53) | 3.05 (1.42) | 3.28 (1.62) |
| Inspiration (4) | 3.12 (1.40) | 3.17 (1.49) | 3.03 (1.58) |
| Inspiration (5) | 3.19 (1.40) | 3.14 (1.38) | 3.11 (1.50) |
| Beauty | 2.12 (1.21) | 2.09 (1.11) | 2.44 (1.42) |
| Boredom (1) | 4.55 (1.16) | 4.39 (1.03) | 4.37 (1.14) |
| Boredom (2) | 4.27 (1.16) | 4.16 (1.22) | 3.98 (1.34) |
| Interest (1) | 1.71 (1.09) | 1.97 (1.09) | 2.03 (1.12) |
| Interest (2) | 1.81 (1.13) | 1.89 (1.10) | 2.13 (1.27) |
| Nostalgia (1) | 2.71 (1.64) | 3.08 (1.56) | 3.05 (1.59) |
| Nostalgia (2) | 1.94 (1.47) | 2.22 (1.27) | 2.18 (1.48) |

Table S3
*Mean and SD values of each variable for each condition.*

*﻿Note.* Standard deviations appear in parentheses.

| Option | Value |
| --- | --- |
| Sampling algorithm | HMC(Hybrid/Hamiltonian Monte Carlo) |
| Number of chains run | 4 |
| Number of steps per chain | 4000 |
| Number of discarded samples | 1000 |

Table S4
*Options for the MCMC procedure.*

*Note.* The source code and the results of the MCMC are available at https://github.com/psychologyKM/VESTA/tree/main/analysis/R.
